# Genetic risk of clozapine-induced leukopenia and neutropenia: a genome-wide association study

**DOI:** 10.1038/s41398-021-01470-z

**Published:** 2021-06-03

**Authors:** Jianhua Chen, Ping Yang, Qian Zhang, Ruirui Chen, Peng Wang, Benxiu Liu, Wensheng Sun, Xuemin Jian, Siying Xiang, Juan Zhou, Ningning Li, Ke Wang, Chengwen Gao, Yanqin Wen, Chuanhong Wu, Jinmai Zhang, Yalin Zhao, Qiangzhen Yang, Meihang Li, Robert Stewart, Yuanchao Sun, Dun Pan, Yujuan Niu, Zhuo Wang, Yifeng Xu, Xingwang Li, Lin He, Zhiqiang Li, Yongyong Shi

**Affiliations:** 1 The Affiliated Hospital of Qingdao University & The Biomedical Sciences Institute of Qingdao University (Qingdao Branch of SJTU Bio-X Institutes), Qingdao University, Qingdao, 266003 PR China; 2 Shanghai Clinical Research Center for Mental Health, Shanghai Key Laboratory of Psychotic Disorders, Shanghai Mental Health Center, Shanghai Jiao Tong University School of Medicine, Shanghai, 200030 PR China; 3 Bio-X Institutes, Key Laboratory for the Genetics of Developmental and Neuropsychiatric Disorders (Ministry of Education), The Collaborative Innovation Center for Brain Science, Shanghai Jiao Tong University, Shanghai, 200030 PR China; 4 Department of Psychological Medicine, Institute of Psychiatry, Psychology & Neuroscience, King’s College London, London, SE5 8AF United Kingdom; 5 Wuhu Fourth People’s Hospital, Wuhu, 242407 PR China; 6 Longquan Mountain Hospital of Guangxi Province, Liuzhou, 545005 PR China; 7 South London and Maudsley NHS Foundation Trust, London, UK; 8 Shanghai Key Laboratory of Sleep Disordered Breathing, Shanghai, 200030 PR China; 9 Shanghai Changning Mental Health Center, 299 Xiehe Road, Shanghai, 200042 PR China; 10 Department of Psychiatry, The First Teaching Hospital of Xinjiang Medical University, Urumqi, 830054 PR China

**Keywords:** Medical genetics, Schizophrenia

## Abstract

**Background:**

Clozapine is considered to be the most effective antipsychotic medication for schizophrenia. However, it is associated with several adverse effects such as leukopenia, and the underlying mechanism has not yet been fully elucidated. The authors performed a genome-wide association study (GWAS) in a Chinese population to identify genetic markers for clozapine-induced leukopenia (CIL) and clozapine-induced neutropenia (CIN).

**Methods:**

A total of 1879 patients (225 CIL cases, including 43 CIN cases, and 1,654 controls) of Chinese descent were included. Data from common and rare single nucleotide polymorphisms (SNPs) were tested for association. The authors also performed a trans-ancestry meta-analysis with GWAS results of European individuals from the Clozapine-Induced Agranulocytosis Consortium (CIAC).

**Results:**

The authors identified several novel loci reaching the threshold of genome-wide significance level (*P* < 5 × 10^−8^). Three novel loci were associated with CIL while six were associated with CIN, and two T cell related genes (*TRAC* and *TRAT1*) were implicated. The authors also observed that one locus with evidence close to genome-wide significance (*P* = 5.08 × 10^−8^) was near the *HLA-B* gene in the major histocompatibility complex region in the trans-ancestry meta-analysis.

**Conclusions:**

The associations provide novel and valuable understanding of the genetic and immune causes of CIL and CIN, which is useful for improving clinical management of clozapine related treatment for schizophrenia. Causal variants and related underlying molecular mechanisms need to be understood in future developments.

## Introduction

Schizophrenia is a severe mental disorder accompanied by considerable morbidity and mortality, which affects ~1% of the population worldwide^1^. The etiology of schizophrenia is still not well understood. Environmental and genetic factors play important roles in the development of schizophrenia^2^. The heritability of schizophrenia is estimated to be 60–80%^2,3^. Antipsychotic medications are commonly used to treat patients with schizophrenia, but responses to these drugs vary widely^4^. Although antipsychotics can relieve symptoms of psychosis, ~75% of patients discontinue their therapy due to adverse effects in two years^5^.

Clozapine is considered to be the most effective antipsychotic medication for schizophrenia and has a distinctive pharmacological profile in treatment-resistant schizophrenia^6^. However, it is also associated with several adverse effects such as weight gain, metabolic dysfunction, cardiovascular disease and leukopenia. Leukopenia is defined as white blood cell (WBC) count less than 4,000 cells per microliter. Neutropenia and agranulocytosis are different types of leukopenia with an absolute neutrophil count (ANC) less than 1,500 and 500 cells per cubic millimeter (mm^−3^), respectively. Clozapine-induced agranulocytosis (CIA) is a severe leukopenia that may be life-threatening, first reported in Finland in 1974^7,8^. Among patients taking clozapine, the cumulative risk of neutropenia is 3.8% and for agranulocytosis is 0.9%^9^.

There is evidence of a genetic contribution in the onset of clozapine-induced leukopenia (CIL), neutropenia (CIN) and CIA^10^; however, the underlying mechanism remains unclear and may involve multiple genes. The HLA (human leukocyte antigen) system, which locates in the major histocompatibility complex (MHC) region, has been reported to be associated with CIL^11–13^. HLA genes such as *HLA-B* and *HLA-DQB1* were reported to be associated with CIL by Clozapine-Induced Agranulocytosis Consortium (CIAC) and many other researchers^11–13^. Previous genome-wide association studies (GWAS) and whole-exome sequencing studies have indicated that other genes such as *SLCO1B3*, *SLCO1B7*, *UBAP2* and *STARD9* also play important roles in the pathophysiology of CIN^12^. Most studies of CIN have focused on individuals of European ancestry. To generalize the results, some recent studies have begun to investigate patients from different ethnic groups. Ancestry-based differences in alleles may be part of the reason for different prevalence of CIL; for example, Duffy-null genotype has been found to be associated with benign neutropenia in patients with African ancestry^14^, and some previously reported SNPs have not been replicated in individuals of Japanese ancestry^12,15^.

Clozapine has been available for Chinese patients since the 1970s. It was once a commonly used antipsychotic drug in China, prescribed for ~25–60% of patients with schizophrenia^16,17^, and the reported prevalence of CIL in China is 3.92% (0.21% for CIA)^16,18^. In this study, we report what we believe to be the first GWAS of CIL in a Chinese population, seeking to identify genetic determinants and provide more information on possible underlying etiology.

## Methods and materials

### Study design and participants

We analyzed genome-wide data of patients with schizophrenia receiving clozapine treatment from a Chinese cohort. Patients were recruited for this study from Wuhu Fourth People’s Hospital and Guangxi Longquan Mountain Hospital from 2007 to 2012. The clinical interviews were conducted by two independent psychiatrists. All patients had diagnoses of schizophrenia according to the Diagnostic and Statistical Manual of Mental Disorders, Fourth Edition (DSM-IV) criteria. Those patients with treatment-resistant schizophrenia^19^ were prescribed clozapine and 1,983 patients were included in this study. Those who developed WBC < 4,000 mm^−3^ during treatment with clozapine were defined as clozapine-induced leukopenia (CIL) cases (*n* = 242). Of these, 46 cases also developed ANC < 1,500 mm^−3^, and were thus defined as clozapine-induced neutropenia (CIN) cases. All controls (*n* = 1,741) received clozapine without developing WBC < 4,000 mm^−3^ or ANC < 1,500 mm^−3^.

We also performed a genome-wide meta-analysis of our Chinese sample with individuals of European ancestry from the Clozapine-Induced Agranulocytosis Consortium (CIAC)^11,12^.

In accordance with the principles in Declaration of Helsinki, the study was approved by the Ethics Committee at the Bio-X Institutes of Shanghai Jiao Tong University and the written informed consent was obtained from each participant. It is confirmed that the research complied with the Guidance of the Ministry of Science and Technology (MOST) for the Review and Approval of Human Genetic Resources.

### Procedures and statistical analysis

Details on genotyping and quality control procedures for the genome-wide analysis of Chinese samples are described in Supplementary Material.

We performed primary genome-wide association analysis for CIL To minimize the effect for population stratification, we performed association analysis separately for two sample sets (according to genetic origin criteria), and then combined the summary in the meta-analysis. For the common variants (minor allele frequency [MAF] ≥ 1%), including both genotyped and imputed variants, association analyses were performed by logistic regression using imputed dosage files and the meta-analysis via METAL with a fixed-effects model. Heterogeneity among the sample collections in the meta-analysis was measured with the I^2^ index and the p value, which was calculated with Cochran’s Q test. For the rare variants, we restricted to the genotyped variants only, association and meta analyses were carried using PLINK with the Cochran–Mantel–Haenszel method.

We conducted secondary analyses on a subset of the more severely affected cases with ANC < 1,500 mm^−3^ (CIN). Controls were still those who received clozapine without developing WBC < 4,000 mm^−3^. Those who had a test result with ANC ≥ 1,500 mm^−3^ and WBC < 4,000 mm^−3^ were excluded from the secondary analysis. All analyses performed were consistent with methods described above for CIL analysis.

We performed the trans-ancestry meta-analysis of our CIN results with data from the CIAG study^11,12^ for common variants. We adopted a fixed-effects model implemented in RICOPILI^20^ for the analysis.

We annotated the genome-wide significant variants and their proxies (*r*^*2*^ ≥ 0.8 in EAS of 1000 Genomes Project Releases Phase 3) to explore putative causal variants and genes underlying these association signals. We also searched for evidence that the common variants were associated with expression of a particular gene in several expression quantitative trait locus (eQTL) datasets: the Genotype-Tissue Expression (GTEx, v7)^21^, immune cells^22^ and lymphoblastoid cell lines (LCLs)^23^. Potential functional effects of the sentinel variants were predicted by the GWAVA (Genome Wide Annotation of VAriants)^24^, a web-based tool to prioritize the functional variations based on a wide range of annotations of noncoding elements (such as ENCODE/GENCODE, evolutionary conservation and GC-content).

### Dual-luciferase reporter assays

An in vitro functional assay was used to investigate allele-dependent effects of the prioritized variants by GWAVA on gene transcription. Experiments were performed with human embryonic kidney (HEK293) cell line. Luciferase reporter constructs were cloned by using the pGL3-promoter vector (Promega, Madison, WI, USA) as a backbone. For each variant, two DNA fragments containing risk or nonrisk allele were produced and all constructs were verified via Sanger sequencing. The pGL3 reporter, risk and nonrisk vectors were transfected into HEK293 cells. Luciferase activities were assessed at 48-h post-transfection according to the manufacturer’s protocols (Promega). The activity of firefly luciferase was normalized to that of Renilla luciferase to control for variations in the transfection efficiency between different wells. All assays were performed in three or more biological replicates in independent experiments, and two-tailed t-tests were performed to analyze statistical differences between experimental groups. Further details are provided in the Supplementary Material.

## Results

**Fig. 1:**
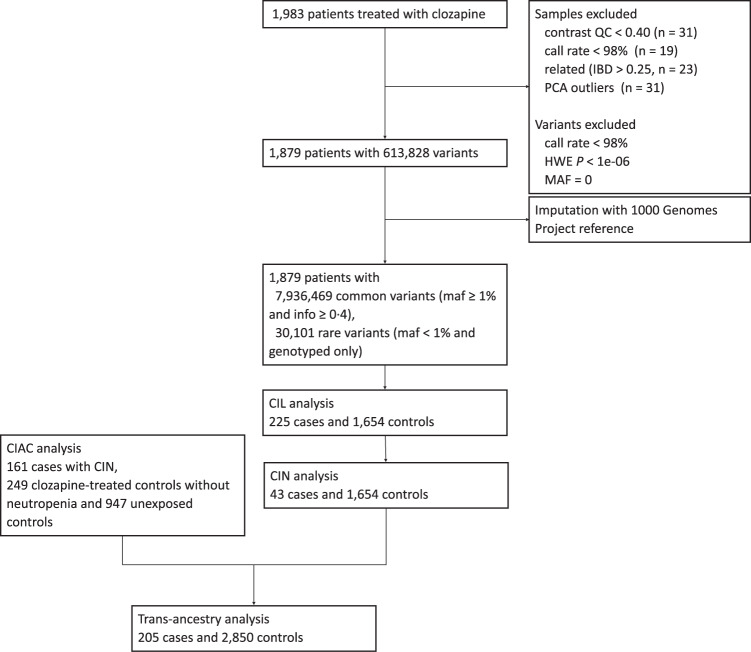
Flowchart for the study design. QC: quality control; IBD: identity-by-descent; PCA: principal component analysis; HWE: Hardy-Weinberg equilibrium; MAF: minor allele frequency; CIL: clozapine induced leukopenia; CIN: clozapine induced neutropenia; CIAC: Clozapine Induced Agranulocytosis Consortium.

**Fig. 2:**
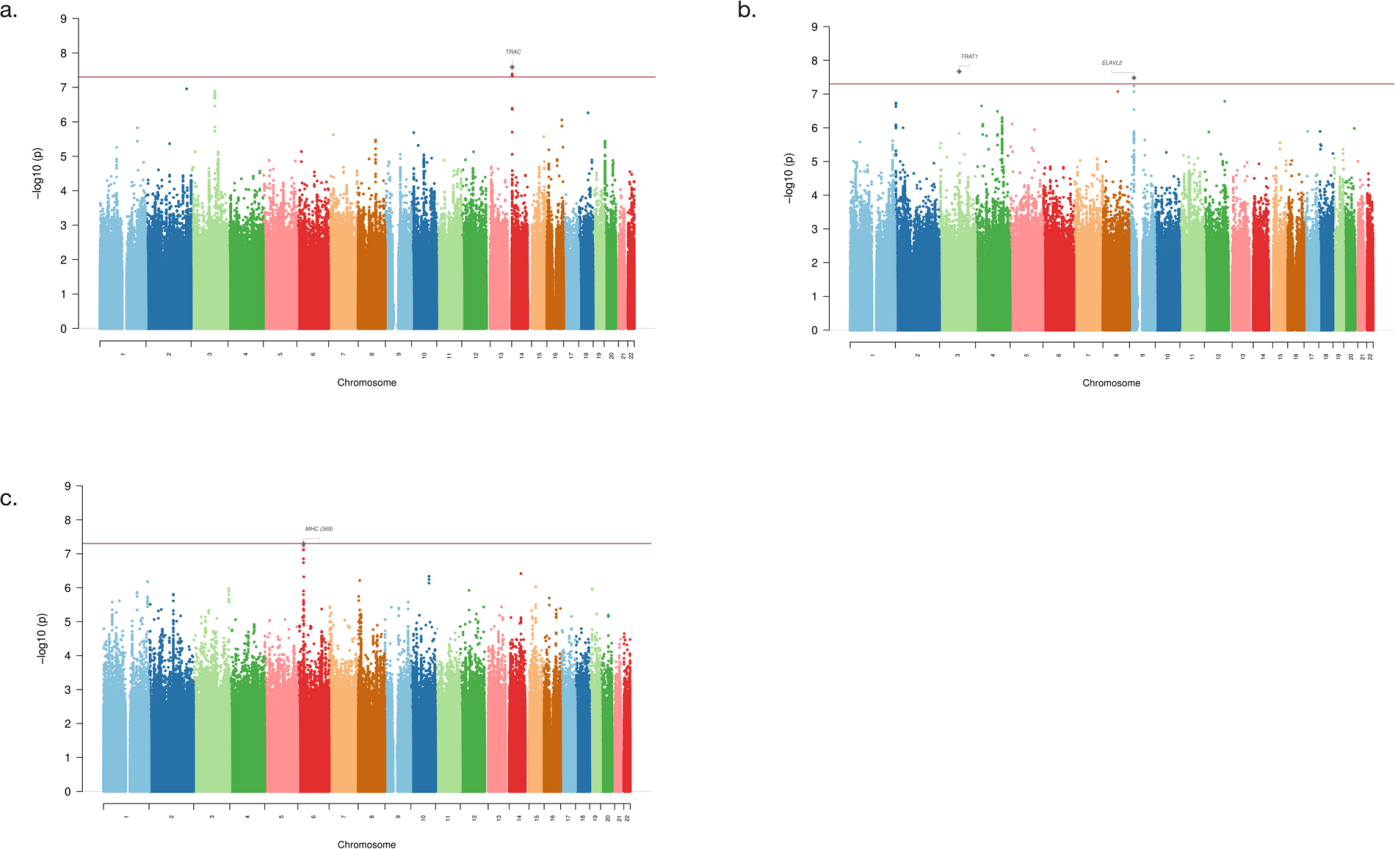
Manhattan plots for GWAS analyses. **a**) the CIL analysis (225 cases and 1,654 controls). **b**) the CIN analysis (43 cases and 1,654 controls). **c**) the trans-ancestry GWAS meta-analysis of CIN (43 cases and 1,654 controls) and CIAC (161 cases and 1,196 controls) datasets. -log10P values for analysis are shown.

From Jan 1, 2007 to Dec 31, 2012, 1,983 patients with treatment-resistant schizophrenia, who were prescribed clozapine, were enrolled from two Mental Health Centers in China for genome-wide genotyping; 104 (5%) failed quality control (see Supplementary Material), of whom 31 did not meet array QC metrics criteria, 19 did not meet call rate criteria, 23 did not meet identity-by-descent criteria, and 31 were excluded as population outliers after a principal component analysis (see Supplementary Fig. 1). The analyzed sample thus comprised 225 CIL cases (mean [±SD] age, 39.3 ± 12.7 years), 43 CIN cases (37.7 ± 13.3 years) and 1,654 controls (36.2 ± 12.7 years)(see Supplementary Table 1). The sample was drawn from two datasets: Dataset 1: Central Chinese, 155 CIL cases (including 18 CIN cases) and 1,355 controls; Dataset 2: Southern Chinese, 70 CIL cases (including 25 CIN cases and 299 controls). 613,828 genotyped SNPs passed the predefined genotyping quality-control criteria for further analysis (see Fig. 1, Supplementary Material). After imputation and quality control, genotypes for 7,966,570 variants were available for downstream analyses. We performed genome-wide association analysis for 7,936,469 common (MAF ≥ 1%, both genotyped and imputed, see Fig. 2) and 30,101 rare (MAF < 1%, genotyped only, see Supplementary Fig. 2) variants.

**Fig. 3:**
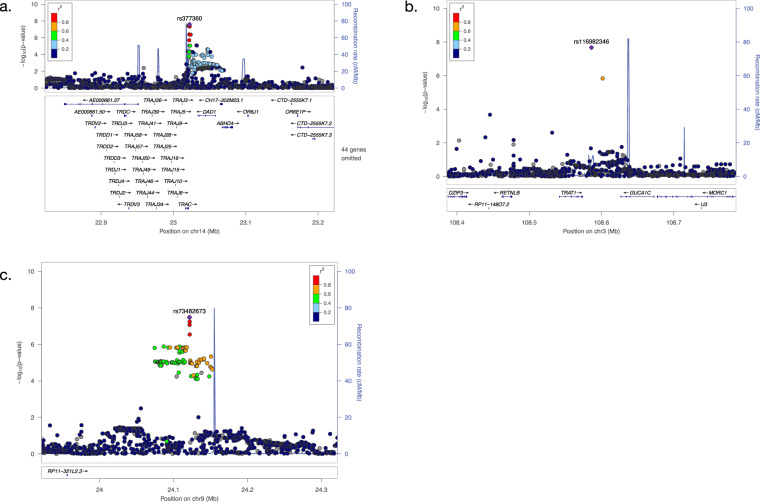
Regional plots for the novel GWS loci. **a**) rs377360. **b**) rs116982346. **c**) rs73482673. -log10P values are shown for SNPs for the region 500 kb on either side of the marker SNPs. The index SNP is shown in purple, and the r2 values of the other SNPs are indicated by color. The r2 values were established on the basis of 1000 Genomes East Asian (EAS) population data (November 2014). The genes within the relevant regions are annotated and shown as arrows.

**Fig. 4:**
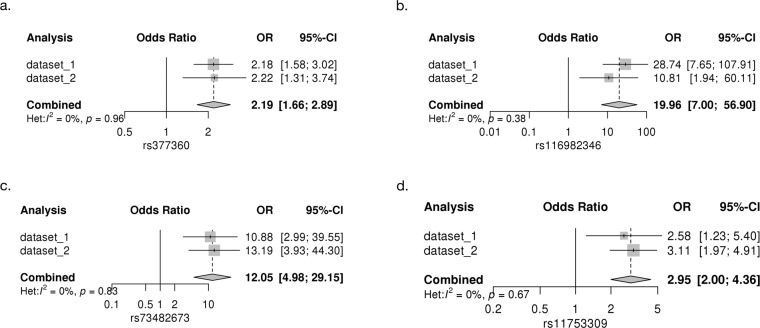
Forest plots for the lead GWS variants in common variant analyses. **a**) rs377360. **b**) rs116982346. **c**) rs73482673. **d**) rs11753309. Area of the square represents the weight of each statistical sample; horizontal lines represent OR and 95% CI in two independent datasets. The diamond represents the total 95% CI estimated in meta-analysis. OR, odds ratio; CI, confidence interval.

In the association analysis of 225 CIL cases and 1,654 controls for common variants, one locus near *TRAC* on chromosome 14 was identified to be associated with CIL genome-wide significantly (five variants, *P* < 5 × 10^−8^, see Fig. 2), and the strongest association was at rs377360 (MAF = 18.9 %, OR = 2.19, 95% CI = 1.66–2.89, *P* = 2.58 × 10^−8^, see Table 1, Fig. 3 and Supplementary Table 2). As shown in the Q-Q plot, the genomic inflation factor (λ = 0.976) indicated good control of population stratification (see Supplementary Fig. 3). None of the genome-wide significant variants and their proxies (*r*^*2*^ ≥ 0.8 in EAS of 1000 Genomes Project Releases Phase 3) fell within coding regions, except rs3701 which overlaps a 3’ untranslated region (3’-UTR) of *TRAC* (see Supplementary Table 3). Based on our eQTL analysis, most were associated with gene expression levels of multiple genes (*P* < 0.05) in whole blood and immune cells (see Supplementary Table 4). The target genes included T cell receptor alpha and delta variable and constant segments coding genes (such as *TRAC*), and also other genes (such as *DAD1*), as shown in Supplementary Table 4. For the rare variants, we identified five genome-wide significant variants from two regions (see Supplementary Fig. 2), which were indexed by rs4773794 in *DCT* on chromosome 13 (MAF = 0.18%, OR = 12.79, *P* = 3.01 × 10^−10^), and rs10512698 in *EEFSEC* on chromosome 3 (MAF = 0.88%, OR = 4.79, *P* = 1.09 × 10^−8^). All these lead variants showed significant associations in both datasets (*P* < 0.05, see Supplementary Table 2) and no evidence for heterogeneity across datasets were observed (Het *P* > 0.05, see Fig. 4 and Supplementary Fig. 4).

**Table 1:** Association results for the identified risk loci from the common variant analyses.

Analysis	CHR	SNP	BP	A1/A2	OR [95% CI]	*P*	Nearby gene
CIL	14	rs377360	23022276	A/T	2.19 [1.66−2.89]	2.58E−08	*TRAC*
CIN	3	rs116982346	108586107	C/G	19.96 [7.00−56.90]	2.15E−08	*TRAT1*
CIN	9	rs73482673	24121611	A/G	12.05 [4.98−29.15]	3.30E−08	*ELAVL2*
TRANS	6	rs11753309	31320645	A/C	2.95 [2.00−4.36]	5.08E−08	*HLA-B*

The CIL analysis is for 225 cases and 1,654 controls (dataset 1: 155 cases and 1,355 controls; dataset 2: 70 cases and 299 controls). The CIN analysis is for 43 cases and 1,654 controls (dataset 1: 18 cases and 1,355 controls; dataset 2: 25 cases and 299 controls). The TRANS analysis is for the trans-ancestry GWAS meta-analysis of CIN analysis (dataset 1: 43 cases and 1,654 controls) and CIAC (dataset 2: 161 cases and 1,196 controls). rs11753309 was close to genome-wide significance.

*CHR* chromosome, *SNP* rs number, *BP* base position based on hg19, *A1/A2* effect allele/other allele, *OR* odds ratio, *95% CI* 95% confidence interval.

For the CIN analysis of 43 cases and 1,654 controls, we identified three associated common variants, having MAF between 1% and 5%, that achieved genome-wide significance. These are from two regions and indexed by rs116982346 (MAF = 1.90%, OR = 19.96, *P* = 2.15 × 10^−8^) on chromosome 3 and rs73482673 (MAF = 1.10%, OR = 12.05, *P* = 3.30 × 10^−8^) on chromosome 9 (see Fig. 3 and Table 1). All these variants showed significant associations in both datasets (*P* < 0.05, see Supplementary Table 2) and no evidence for heterogeneity across datasets was observed (Het *P* > 0.05, see Fig. 4). All the genome-wide significant variants and their proxies are in intergenic regions, and the nearest coding genes are *TRAT1* (rs116982346) and *ELAVL2* (rs73482673)(see Fig. 4). One of them was associated with gene expression level of *ELAVL2* in lymphoblastoid cell lines at *P* < 0.05 (see Supplementary Table 4). We also found several CIN associated rare variants (see Supplementary Fig. 2), which were indexed by rs9808117 in *HECW2* on chromosome 2 (MAF = 0.64%, OR = 9.10, *P* = 9.82 × 10^−9^), rs373695 in *F13A1* on chromosome 6 (MAF = 0.06%, OR = 74.83, *P* = 1.36 × 10^−8^), rs7501702 ear *MFAP4* on chromosome 17 (MAF = 0.33%, OR = 14.95, *P* = 1.76 × 10^−8^) and rs8024434 in *NEO1* on chromosome 15 (MAF = 0.36%, OR = 19.39, *P* = 4.83 × 10^−8^)(see Supplementary Table 2). Some proxies of the genome-wide significant variants are variants in coding regions, such as 3’-UTR in *EPN2* and a missense variant in *B9D1* for rs7501702, 3’-UTR in *REC114* and a synonymous variant in *HCN4* for rs8024434 (see Supplementary Table 3).

To explore the effects of power and heterogeneity, we performed a trans-ancestry GWAS meta-analysis of CIAC (161 cases and 1,196 controls) with CIN analysis (43 cases and 1,654 controls) for common variants. The combined GWAS meta-analysis identified rs11753309 (MAF > 0.05) near *HLA-B* on chromosome 6 as close to genome-wide significant (OR = 2.95, 95% CI = 2.00–4.36; *P* = 5.08 × 10^−8^, Table 1) and no evidence for heterogeneity across ancestries was observed (*P* > 0.05)(see Fig. 4).

**Fig. 5:**
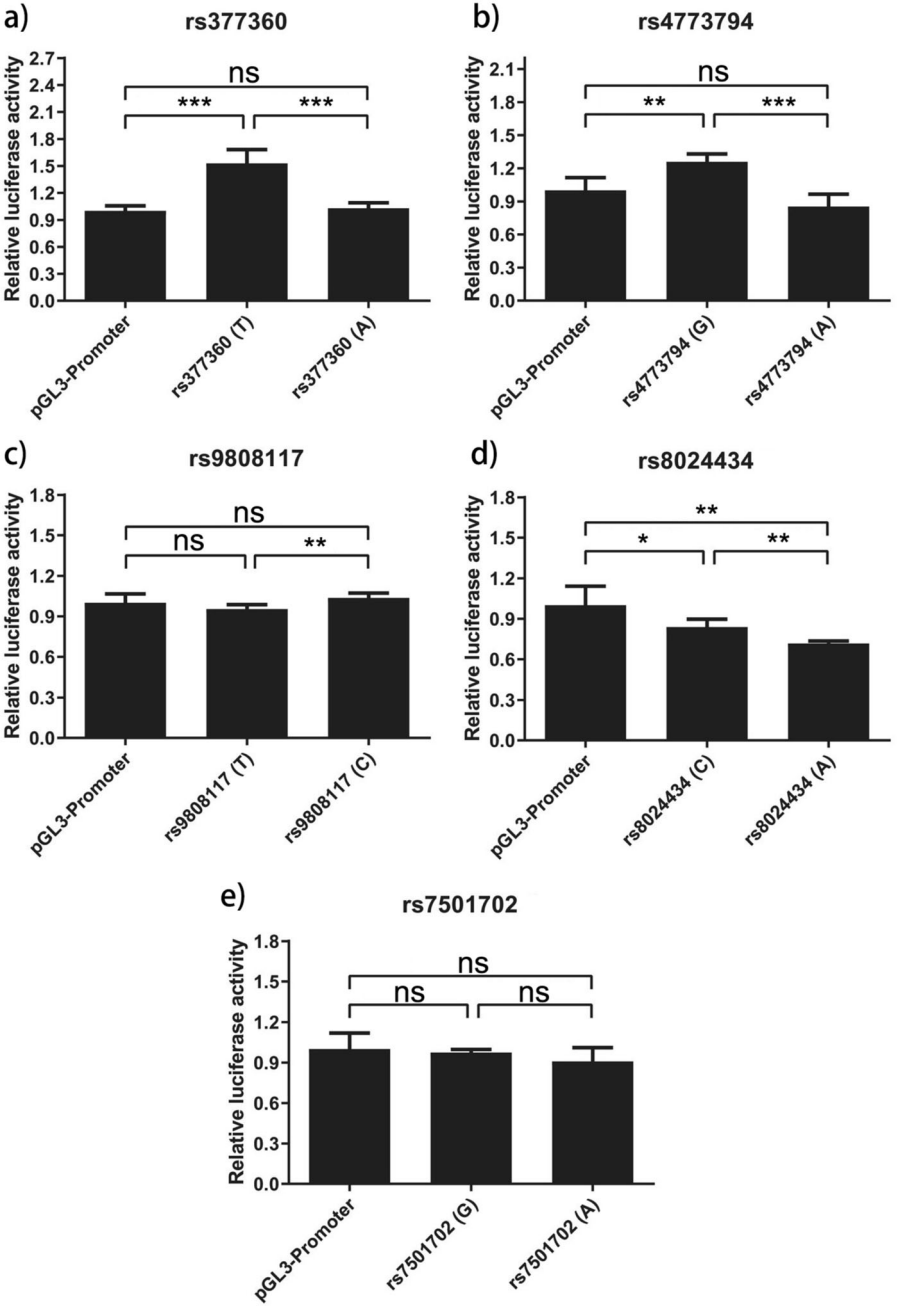
Luciferase reporter assays comparing transcriptional activation with pGL3-Promoter or variant alleles of genetic variants. **a**) rs377360. **b**) rs4773794. **c**) rs9808117. **d**) rs8024434. **e**) rs7501702. HEK-293 cells were transfected with luciferase reporter constructs for both alleles of each variant. Each construct was transfected five times and assayed in quintuplicate in each experiment. The luciferase activities, normalized with a cotransfected Renilla activities, were expressed by taking the normalized luciferase activity of the vector (pGL3-Promoter) to be 1. Data represent means±SDs. P values were determined by using the t-test. Significance indicators will be written above the bars: *** if p < 0·001, ** if p < 0·01, * if p < 0·05,ns otherwise.

Five prioritized variants (rs377360 and rs4773794 for CIL; rs9808117, rs8024434 and rs7501702 for CIN) by their highest GWAVA scores (see Supplementary Table 5) were tested for transcriptional activity using dual-luciferase reporter assay (see Supplementary Table 6). Luciferase assays showed the DNA segment containing rs377360-T (risk for CIL) increased transcriptional activity in HEK293 cells (49% increase [SD 16.6] comparing to nonrisk allele, *P* < 0.001, Fig. 5). Consistent with GWAVA prediction, we found rs4773794-G (protective for CIL) was associated with an increase of transcriptional activity in HEK293 cells most significantly (50% increase [SD 27.2] comparing to risk allele, *P* < 0.001, Fig. 5). For the CIN associated variants, mild or little affections on transcriptional activity were observed (see Fig. 5).

## Discussion

To our knowledge, our study is the first GWAS of CIL and CIN in individuals of Chinese ancestry and identified nine novel genome-wide significant loci. Five of the nine index variants identified in our GWAS mapped to the intron of a protein-coding gene, and all except one of the others had a protein-coding gene within 15 kb. The trans-ancestry meta-analysis of the Chinese and European ancestry identified one risk locus which was close to genome-wide significance.

Mechanisms underlying CIL and CIN have not yet been fully elucidated, although an immune reaction has been hypothesized for a long time as implicated^10^. We identified two T cell related genes in this study, *TRAC* (T cell receptor alpha constant) and *TRAT1* (T Cell Receptor Associated Transmembrane Adaptor 1). The *TRAC* gene locating on 14q11-12 encodes the constant region of the T cell receptor (TCR) alpha chain. The alpha loci are synthesized by variable, joining, and constant segments. TCR recognizes antigen fragments as peptides bound to MHC molecules, which are important for thymic selection. Deficiency of MHC may cause abnormal immune function and result in a lack of maturation of the corresponding T cells^25^. Diseases associated with *TRAC* include TCR alpha/beta deficiency. It has been reported that *TRAC* mutations cause a human immunodeficiency disorder characterized by a lack of TCR αβ + T cells^26^. *TRAT1* also plays an important role in the immune process. Among the predominantly downregulated cell surface antigens, TRAT1 along with ZAP70, LCK and TCR associated tyrosine kinases are involved in TCR activation, and were all simultaneously downregulated^27^.

Although we did not identify susceptibility loci reaching the level of genome-wide significance after the trans-ancestry meta-analysis, we found a near-significant risk locus for CIN near *HLA-B*. HLA-B is a paralog of HLA class I heavy chain, which is anchored in the membrane and plays an important role in the immune system by presenting peptides derived from the lumen of the endoplasmic reticulum. HLA-B was reported as a susceptibility gene of CIA in Caucasian population^11^, while HLA-B*59:01 was suggested as a risk factor for CIA in the Japanese population^28^. Most studies of CIL have been conducted in Caucasian populations, and it may not be possible to replicate identified variants across different races and populations for many reasons. However, associations of variants from GWAS have generally added evidence for immune-related mechanisms.

The biological activation or the conversion of stable metabolites to chemically reactive nitrogen ions could represent another causal pathway^29–31^. However, hypotheses of toxicity and immune regulation are not necessarily mutually exclusive. The combination of these reactive metabolites can result in CIL, CIN or CIA through direct toxicity or via initiation of immune mechanisms or both. Clozapine may potentially be oxidized into toxic nitrenium ions, which bind to neutrophils and cause an autoimmune reaction or induce cell death.

In this study, we also found several other compelling candidate genes. *NEO1* at 15q24.1 encodes a cell surface protein composed of four N-terminal immunoglobulin-like domains, which may be involved in cell growth and differentiation and cell adhesion. Neo1 contributes to the inflammatory response and resolution mechanisms^32^. Genetic deletion or functional inhibition of Neo1 leads to reduced neutrophil recruitment and shortened the neutrophil lifespan by increasing apoptosis^33^. *DCT* at 13q32.1 encodes dopachrome tautomerase and participates in melanocytes pigment biosynthesis with tyrosinase and tyrosinase-related protein 1^34^. Meanwhile it also plays a role in oxidative stress and apoptotic stimuli respondence^35,36^. An experiment in mice found that the spatiotemporal distribution of *DCT* expression was related to cortical neurogenesis during embryonic development^37^. Diseases associated with *DCT* include microphthalmia and melanoma. Previous studies have proved that a large portion of chromosome, spanning 68 Mb from 13q12 to 13q34, could be linked to susceptibility to schizophrenia^38,39^. *EEFSEC* located at 3q21.3, encoding selenocysteine-tRNA specific eukaryotic elongation factor, which is essential for the synthesis of selenoproteins^40^. Selenoproteins contain several proteins with different functions, some of which play important roles in antioxidant defense preservation and anti-inflammatory regulation^41^. Polymorphisms or mutations in *EEFSEC* could account for a variety of disorders, including neuropsychiatric disorders and immune dysfunctions^42^. We hypothesize that *EEFSEC* may participate in the mechanism of CIL through selenoproteins. Many selenoproteins participate in redox signaling, redox homeostasis, and antioxidant defense by the action of glutathione peroxidase^43^. The amount of selenoproteins and glutathione peroxidase decreases under oxidative stress conditions, and the activity of selenium-dependent glutathione peroxidase has been found to be lower in patients treated with clozapine^44^. Activation of clozapine and/or its metabolites can produce electrophilic nitrenium ions, which might either covalently bind to neutrophils such as glutathione to cause cell death or cause oxidative stress-induced neutrophil apoptosis^45,46^.

We feel it is reasonable to assume that the risks of CIL and CIN are genetically complex, involving several genes. The application of GWAS and other modern genomic technologies gives us a better understanding of the genetic basis of CIL and CIN. Our study is the first GWAS of CIL and CIN in individuals of Chinese ancestry and identified several novel genome-wide significant loci. However, there are still some limitations in our study. First, many SNPs detected were noncoding variants with unknown effects, which is a common problem with the GWAS approach. There may also be potential Winner’s curse bias in estimating the average genetic effect of a set of rare variants and identifying statistically significant associations. Therefore, further functional studies are needed to verify the related biological mechanisms. Second, since some conditions such as circadian rhythm can affect the amount of peripheral blood neutrophils in patients, other related factors should also be taken into account. Finally, an important challenge is that there is relatively rare incidence of CIN and even rarer incidence of CIA in the Chinese population. In fact, only six CIA cases met the criteria in our study, which may be due to the success of the monitoring system, since once leukopenia is detected, clozapine will be discontinued. It is therefore impossible to know which patients would continue to develop CIA. The rare incidence limits the availability of suitable patients and thus the sample size for research. Therefore, replication of larger sample sizes is critical for clinical applications.

Clozapine is the only effective medication for treatment-resistant schizophrenia, but its use is limited due to the risk of leukopenia, particularly agranulocytosis. Genetic and immune factors are likely to play an important role in determining risk for such adverse reactions. Our results provide novel and valuable understanding of the genetic and immune causes of CIL and CIN. Causal variants and related underlying molecular mechanisms need to be understood in future developments, which will be key to improving diagnostic and therapeutic capabilities in treatment-resistant schizophrenia.

## Supplementary information

Supplementary Material

## References

[1] Kahn RS (2015). Schizophrenia. Nat. Rev. Dis. Prim.

[2] Burmeister M, McInnis MG, Zollner S (2008). Psychiatric genetics: progress amid controversy. Nat. Rev. Genet.

[3] Nothen MM, Nieratschker V, Cichon S, Rietschel M (2010). New findings in the genetics of major psychoses. Dialogues Clin. Neurosci.

[4] Huber CG, Naber D, Lambert M (2008). Incomplete remission and treatment resistance in first-episode psychosis: definition, prevalence and predictors. Expert Opin. Pharmacother.

[5] Leucht S, Heres S (2006). Epidemiology, clinical consequences, and psychosocial treatment of nonadherence in schizophrenia. J. Clin. Psychiatry.

[6] Kane J, Honigfeld G, Singer J, Meltzer H (1988). Clozapine for the treatment-resistant schizophrenic. A double-blind comparison with chlorpromazine. Arch. Gen. Psychiatry.

[7] Griffith RW, Saameli K (1975). Letter: Clozapine and agranulocytosis. Lancet.

[8] Idanpaan-Heikkila J, Alhava E, Olkinuora M, Palva I (1975). Letter: Clozapine and agranulocytosis. Lancet.

[9] Myles N (2018). Meta-analysis examining the epidemiology of clozapine-associated neutropenia. Acta Psychiatr. Scand.

[10] Legge SE, Walters JT (2019). Genetics of clozapine-associated neutropenia: recent advances, challenges and future perspective. Pharmacogenomics.

[11] Goldstein JI (2014). Clozapine-induced agranulocytosis is associated with rare HLA-DQB1 and HLA-B alleles. Nat. Commun.

[12] Legge SE (2017). Genome-wide common and rare variant analysis provides novel insights into clozapine-associated neutropenia. Mol. Psychiatry.

[13] Valevski A (1998). HLA-B38 and clozapine-induced agranulocytosis in Israeli Jewish schizophrenic patients. Eur. J. Immunogenet.

[14] Legge SE (2019). A genome-wide association study in individuals of African ancestry reveals the importance of the Duffy-null genotype in the assessment of clozapine-related neutropenia. Mol. Psychiatry.

[15] Saito T (2017). Transethnic replication study to assess the association between clozapine-induced agranulocytosis/granulocytopenia and genes at 12p12.2 in a Japanese Population. Biol. Psychiatry.

[16] Si TM (2012). Use of clozapine for the treatment of schizophrenia: findings of the 2006 research on the china psychotropic prescription studies. Clin. Psychopharmacol. Neurosci.

[17] Tang YL (2008). Clozapine in China. Pharmacopsychiatry.

[18] de With SAJ, Pulit SL, Staal WG, Kahn RS, Ophoff RA (2017). More than 25 years of genetic studies of clozapine-induced agranulocytosis. Pharmacogenomics J.

[19] Kane, J. M. et al. Clinical guidance on the identification and management of treatment-resistant schizophrenia. *J Clin Psychiatry* **80**, 18com12123 (2019). 10.4088/JCP.18com12123 30840788

[20] Lam M (2020). RICOPILI: rapid imputation for COnsortias PIpeLIne. Bioinformatics.

[21] Carithers LJ, Moore HM (2015). The Genotype-Tissue Expression (GTEx) Project. Biopreservation Biobanking.

[22] Ishigaki K (2017). Polygenic burdens on cell-specific pathways underlie the risk of rheumatoid arthritis. Nat. Genet..

[23] Grundberg E (2012). Mapping cis- and trans-regulatory effects across multiple tissues in twins. Nat. Genet.

[24] Ritchie GRS, Dunham I, Zeggini E, Flicek P (2014). Functional annotation of noncoding sequence variants. Nat. Methods.

[25] La Gruta NL, Gras S, Daley SR, Thomas PG, Rossjohn J (2018). Understanding the drivers of MHC restriction of T cell receptors. Nat. Rev. Immunol.

[26] Morgan NV (2011). Mutation in the TCRalpha subunit constant gene (TRAC) leads to a human immunodeficiency disorder characterized by a lack of TCRalphabeta+ T cells. J. Clin. Invest.

[27] Wang M, Windgassen D, Papoutsakis ET (2008). Comparative analysis of transcriptional profiling of CD3+, CD4+ and CD8+ T cells identifies novel immune response players in T-cell activation. BMC Genomics.

[28] Saito T (2016). Pharmacogenomic study of clozapine-induced agranulocytosis/granulocytopenia in a Japanese population. Biol. Psychiatry.

[29] Flanagan RJ, Dunk L (2008). Haematological toxicity of drugs used in psychiatry. Hum. Psychopharmacol.

[30] Maggs JL, Williams D, Pirmohamed M, Park BK (1995). The metabolic formation of reactive intermediates from clozapine, a drug associated with agranulocytosis in man. J. Pharm. Exp. Ther.

[31] Pirmohamed M, Park K (1997). Mechanism of clozapine-induced agranulocytosis: current status of research and implications for drug development. CNS Drugs.

[32] Ravichandran KS (2011). Beginnings of a good apoptotic meal: the find-me and eat-me signaling pathways. Immunity.

[33] Schlegel M (2018). Inhibition of neogenin fosters resolution of inflammation and tissue regeneration. J. Clin. Invest.

[34] Abdel-Malek Z (1993). Contribution of melanogenic proteins to the heterogeneous pigmentation of human melanocytes. J. Cell Sci.

[35] Ainger SA (2014). DCT protects human melanocytic cells from UVR and ROS damage and increases cell viability. Exp. Dermatol.

[36] Sendoel A, Kohler I, Fellmann C, Lowe SW, Hengartner MO (2010). HIF-1 antagonizes p53-mediated apoptosis through a secreted neuronal tyrosinase. Nature.

[37] Jiao Z (2006). Dopachrome tautomerase (Dct) regulates neural progenitor cell proliferation. Dev. Biol.

[38] Brzustowicz LM (1999). Linkage of familial schizophrenia to chromosome 13q32. Am. J. Hum. Genet.

[39] Detera-Wadleigh SD, McMahon FJ (2006). G72/G30 in schizophrenia and bipolar disorder: review and meta-analysis. Biol. Psychiatry.

[40] Dobosz-Bartoszek M (2016). Crystal structures of the human elongation factor eEFSec suggest a non-canonical mechanism for selenocysteine incorporation. Nat. Commun.

[41] Labunskyy VM, Hatfield DL, Gladyshev VN (2014). Selenoproteins: molecular pathways and physiological roles. Physiol. Rev.

[42] Hornig M (2013). The role of microbes and autoimmunity in the pathogenesis of neuropsychiatric illness. Curr. Opin. Rheumatol.

[43] Touat-Hamici Z, Legrain Y, Bulteau AL, Chavatte L (2014). Selective up-regulation of human selenoproteins in response to oxidative stress. J. Biol. Chem.

[44] Miljevic C (2010). Lipid status, anti-oxidant enzyme defence and haemoglobin content in the blood of long-term clozapine-treated schizophrenic patients. Prog. Neuropsychopharmacol. Biol. Psychiatry.

[45] Husain Z (2006). Increased FasL expression correlates with apoptotic changes in granulocytes cultured with oxidized clozapine. Toxicol. Appl Pharm.

[46] Ip J, Wilson JX, Uetrecht JP (2008). Testing the hypothesis that vitamin C deficiency is a risk factor for clozapine-induced agranulocytosis using guinea pigs and ODS rats. Chem. Res Toxicol.

