# Correction to: Genetic risk of clozapine-induced leukopenia and neutropenia: a genome-wide association study

**DOI:** 10.1038/s41398-021-01484-7

**Published:** 2021-06-30

**Authors:** Jianhua Chen, Ping Yang, Qian Zhang, Ruirui Chen, Peng Wang, Benxiu Liu, Wensheng Sun, Xuemin Jian, Siying Xiang, Juan Zhou, Ningning Li, Ke Wang, Chengwen Gao, Yanqin Wen, Chuanhong Wu, Jinmai Zhang, Yalin Zhao, Qiangzhen Yang, Meihang Li, Robert Stewart, Yuanchao Sun, Dun Pan, Yujuan Niu, Zhuo Wang, Yifeng Xu, Xingwang Li, Lin He, Zhiqiang Li, Yongyong Shi

**Affiliations:** 1 The Affiliated Hospital of Qingdao University & The Biomedical Sciences Institute of Qingdao University (Qingdao Branch of SJTU Bio-X Institutes), Qingdao University, Qingdao, 266003 PR China; 2 Shanghai Clinical Research Center for Mental Health, Shanghai Key Laboratory of Psychotic Disorders, Shanghai Mental Health Center, Shanghai Jiao Tong University School of Medicine, Shanghai, 200030 PR China; 3 Bio-X Institutes, Key Laboratory for the Genetics of Developmental and Neuropsychiatric Disorders (Ministry of Education), The Collaborative Innovation Center for Brain Science, Shanghai Jiao Tong University, Shanghai, 200030 PR China; 4 Department of Psychological Medicine, Institute of Psychiatry, Psychology & Neuroscience, King’s College London, London, SE5 8AF UK; 5 Wuhu Fourth People’s Hospital, Wuhu, 242407 PR China; 6 Longquan Mountain Hospital of Guangxi Province, Liuzhou, 545005 PR China; 7 South London and Maudsley NHS Foundation Trust, London, UK; 8 Shanghai Key Laboratory of Sleep Disordered Breathing, Shanghai, 200030 PR China; 9 Shanghai Changning Mental Health Center, 299 Xiehe Road, Shanghai, 200042 PR China; 10 Department of Psychiatry, The First Teaching Hospital of Xinjiang Medical University, Urumqi, 830054 PR China

**Keywords:** Medical genetics, Schizophrenia

Correction to: *Translational Psychiatry* 10.1038/s41398-021-01470-z, published online 3 June 2021

The original version of this article unfortunately contained a mistake. The following authors all contributed equally to this article: Jianhua Chen, Ping Yang, Qian Zhang and Ruirui Chen. We apologize for the mistake. The original article has been corrected.

