# Supplementary material for: Genetic risk of clozapine-induced leukopenia and neutropenia: a genome-wide association study
Source: Transl Psychiatry. 2021 Jun 3;11:343. doi: 10.1038/s41398-021-01470-z (PMC8175348; doi:10.1038/s41398-021-01470-z)
Supplement: Supplementary file 1 — Supplementary Material [file 41398_2021_1470_MOESM1_ESM.docx]

**Supplementary Material**

**Genotyping,** **quality control (QC) and genotype imputation**

Genome-wide genotyping was performed at the Affymetrix Genome-Wide Human SNP Array 6.0 (SNP 6.0) platform (Santa Clara, CA, USA) in this study. Genotyping Console™ software was used to create genotype calls for collections of CEL files using the Birdseed v2 algorithms. A sencond genotype calling method from Affymetrix Axiom™ Analysis Suite was also adopted according to SNP 6.0's best practice workflow. The concordance rate between two algorithms was ~99.4%, and we restriced to the concordant genotypes for further analysis.

Raw data were quality controlled within two layers: individuals and genotypes. For the individuals, the screening strategy was as follows: those showing opposite self-reported gender with genotype gender were excluded, those with low genotyping call rates (<95% of the overall SNPs) were excluded, and those with lower call rates showing hidden relatedness in pairwise descent analysis (PI_HAT>0.25 by PLINK[^1^](#_ENREF_1)) were also excluded. Then, the genotype filter was implemented: SNPs with call rates <95%, MAF = 0% or with significant HWE deviation (p<1×10^‑6^) in the control cohort were excluded.

After applying QC standards, 613,828 SNPs for 1,879 individuals were performed for imputation (225 CIL cases, including 43 CIN cases, and 1,654 controls).

The genotypes were used for further analyses (including genome-wide phasing and imputation, association analysis and meta-analysis) using RICOPILI.[^2^](#_ENREF_2) The 1000 Genomes Project data set (release v3, URLs) was used as reference panel.[^3^](#_ENREF_3)

**Analysis of population substructure**

A linkage disequilibrium (LD)-based SNP pruning analysis was performed with PLINK[^1^](#_ENREF_1) using parameter “--indep-pairwise 50 10 0.2”. Using the LD-pruned autosomal SNP genotypes, principal component analysis (PCA) was conducted to evaluate the population substructure of the sample using EIGENSTRAT software[^4^](#_ENREF_4). Population outliers (deviated from the mean on one of the top 10 inferred axes of variation more than 6 s.d.) were removed.

**Materials for *in vitro* functional assay**

The pGL3-promoter and pRL-TK reporter plasmids, Dual-Luciferase® Reporter Assay System were purchased from Promega (USA). The FastDigest *Xho*I and *Kpn*I were obtained from Thermo Scientific (Lithuania) and T4 DNA Ligase was from New England Biolabs (USA). Dulbecco’s Modified Eagle’s Medium (DMEM) and fetal bovine serum (FBS) were obtained from Biological Industries (Israel). Penicillin-streptomycin (10,000 U/mL penicillin and 10 mg/mL streptomycin) and trypsin with EDTA were obtained from Procell Life Science&Technology Co,.Ltd. (China). The XfectTM Transfection Reagent was purchased from TaKaRa Bio (China).

**Cell culture**

Human embryonic kidney cell line HEK 293 was obtained from Huaxi hospital (China), and was cultured in DMEM supplemented with 10% heat-inactivated FBS, and 1% penicillin-streptomycin in a humidified atmosphere at 37˚C with 5% CO^2^.

**Luciferase reporter vectors construction**

Luciferase reporter vectors were constructed using the pGL3-promoter vector as a backbone. Two complementary oligonucleotides around each SNP were designed and a sense and antisense oligonucleotide were annealed to form a 31-bp DNA fragment with a *Kpn*I site and a *Xho*I site at the 5’ and 3’ end, respectively (see Supplementary Table 6). The DNA fragments were cloned into the *Kpn*I/*Xho*I sites upstream of the SV40 promoter in the pGL3-promoter reporter plasmid. Both allele constructs for each SNP were generated and all constructs were verified by DNA sequencing. The pRL-TK vector was used as an internal control.

**Dual luciferase reporter assays**

HEK 293 cells were plated in 24-well plates and cultured at 50-70% confluence for 12 hours, and then transfected with a pGL3 reporter contruct and the pRL-TK vector at a ratio of 19:1 (590 ng of total plasmid per cell) by using the X-fect transfection reagent.

Luciferase activity was assayed 48 hours after transfection. Cells were washed with PBS and lysed with 100 μL Passive Lysis Buffer (PLB) in each well. After shaking for 15 minutes at room temperature, the cell lysates were collected. Both firefly and renilla luciferase activities were measured in the same sample by using the Dual-Glo Luciferase Assay System. 10 μL cell lysate was added into 50 μL LAR II working solution and the luminescence of the samples were read immediately for 2s as firefly luciferase activities. Then 50 μL Stop&Glo® Reagent was added into the mixture and the luminescence of the samples were read immediately for 2s as renilla luciferase activities. The firefly luciferase activity was normalized to the renilla luciferase activity of the same sample. At least three replicate samples were used in all transfections, and experiments were repeated three times.

**References**

1. Chang CC, Chow CC, Tellier LCAM, Vattikuti S, Purcell SM, Lee JJ. Second-generation PLINK: rising to the challenge of larger and richer datasets. *Gigascience* 2015; **4**(1): 7.

2. Lam M, Awasthi S, Watson HJ, et al. RICOPILI: Rapid Imputation for COnsortias PIpeLIne. *Bioinformatics* 2020; **36**(3): 930-3.

3. Genomes Project C, Abecasis GR, Auton A, et al. An integrated map of genetic variation from 1,092 human genomes. *Nature* 2012; **491**(7422): 56-65.

4. Price AL, Patterson NJ, Plenge RM, Weinblatt ME, Shadick NA, Reich D. Principal components analysis corrects for stratification in genome-wide association studies. *Nature Genetics* 2006; **38**(8): 904-9.

**Supplementary Figure 1:** Principal component analysis of the studied cohorts.

a) Dataset 1: Central Chinese

b) Dataset 2: Southern Chinese

Principal components analysis was performed using the LD-pruned genome-wide SNP genotypes of the studied cohorts (cases shown in blue and controls shown in red).

**Supplementary Figure 2:** Manhattan plots of the GWAS analyses for rare variants.

a) CIL analysis (225 cases and 1,654 controls)

b) CIN analysis (43 cases and 1,654 controls)

-log_10_P values for analysis are shown.

**Supplementary Figure 3:** Quantile-quantile (QQ) plots of the GWAS analyses.

a) CIL analysis (225 cases and 1,654 controls)

b) CIN analysis (43 cases and 1,654 controls)

c) Trans-ancestry meta-analysis of CIAC (161 cases and 1,196 controls) and CIN (43 cases and 1,654 controls)

**Supplementary Figure 4:** Forest plots

Area of the square represents the weight of each statistical sample; horizontal lines represent OR and 95% CI in two independent datasets. The diamond represents the total 95% CI estimated in meta-analysis. OR, odds ratio; CI, confidence interval.

**Supplementary Table 1:** Description of the GWAS samples

| **Group** | **n (dataset1/dataset2)** | **Age (years, mean±sd)** | **Female (n)** |
| --- | --- | --- | --- |
| CIL | 225 (155/70) | 39.3±12.7^b^ | 132 |
| CIN | 43 (18/25) | 37.7±13.3^a^ | 23 |
| Control | 1654 (1355/299) | 36.2±12.7 | 637 |

CIL: patients developed WBC < 4,000 mm^−3^ during treatment with clozapine. CIN: those developed ANC < 1,500 mm^−3^. Control: those without developing WBC < 4,000 mm^−3^ or ANC < 1,500 mm^−3^. dataset1: Central Chinese. dataset2: Southern Chinese. ^a^No difference compared with the Control group (*P* > 0.05), ^b^*P* < 0.05 compared with the Control group.

**Supplementary Table 2:** Results for the index SNPs in the genomic loci that reached genome-wide significance

| **Analysis** | **CHR** | **SNP** | **BP** | **A1/A2** | **Meta-analysis** | | **Dataset 1** | | | | **Dataset 2** | | | |
| --- | --- | --- | --- | --- | --- | --- | --- | --- | --- | --- | --- | --- | --- | --- |
|  |  |  |  |  | **OR**  **[95% CI]** | **P** | **F_A** | **F_U** | **OR**  **[95% CI]** | **P** | **F_A** | **F_U** | **OR**  **[95% CI]** | **P** |
| CIL | 14 | rs377360 | 23022276 | A/T | 2.19  [1.66-2.89] | 2.58E-08 | 28.57% | 18.76% | 2.18  [1.58-3.02] | 2.54E-06 | 29.28% | 19.67% | 2.22  [1.31-3.74] | 2.89E-03 |
| CIL | 3 | rs10512698 | 127965570 | G/T | 4.79  [2.64-8.67] | 1.09E-08 | 4.22% | 0.81% | 5.38  [2.68-10.79] | 1.21E-07 | 4.29% | 1.17% | 3.78  [1.25-11.43] | 1.17E-02 |
| CIL | 13 | rs4773794 | 95121488 | A/G | 12.79  [4.56-35.87] | 3.01E-10 | 2.58% | 0.18% | 14.33  [4.66-44.08] | 1.03E-09 | 1.43% | 0.17% | 8.65  [0.78-96.03] | 3.47E-02 |
| CIN | 3 | rs116982346 | 108586107 | C/G | 19.96  [7.00-56.90] | 2.15E-08 | 14.35% | 1.94% | 28.74  [7.65-107.91] | 6.52E-07 | 6.58% | 1.92% | 10.81  [1.94-60.11] | 6.55E-03 |
| CIN | 9 | rs73482673 | 24121611 | A/G | 12.05  [4.98-29.15] | 3.30E-08 | 9.07% | 1.06% | 10.88  [2.99-39.55] | 2.89E-04 | 11.55% | 1.20% | 13.19  [3.93-44.30] | 2.98E-05 |
| CIN | 2 | rs9808117 | 197110855 | T/C | 9.10  [3.58-23.15] | 9.82E-09 | 8.33% | 0.48% | 18.85  [5.13-69.26] | 7.85E-10 | 8.00% | 1.34% | 6.41  [1.86-22.10] | 7.89E-04 |
| CIN | 6 | rs373695 | 6184352 | G/A | 74.83  [6.15-910.70] | 1.36E-08 | 2.78% | 0.07% | 38.66  [3.42-436.72] | 1.08E-06 | 2.00% | 0.00% | n.a.  [n.a.-n.a.] | 5.38E-04 |
| CIN | 15 | rs8024434 | 73547727 | C/A | 19.39  [4.76-78.94] | 4.83E-08 | 5.56% | 0.44% | 13.20  [2.85-61.24] | 1.93E-05 | 2.00% | 0.00% | n.a.  [n.a.-n.a.] | 5.38E-04 |
| CIN | 17 | rs7501702 | 19293727 | A/G | 14.95  [3.89-57.41] | 1.76E-08 | 8.33% | 0.37% | 24.55  [6.46-93.30] | 4.65E-12 | 0.00% | 0.17% | n.a.  [n.a.-n.a.] | 7.72E-01 |
| TRANS | 6 | rs11753309 | 31320645 | A/C | 2.95  [2.00-4.36] | 5.08E-08 | 13.80% | 7.00% | 2.58  [1.23-5.40] | 1.18E-02 | 14.75% | 5.85% | 3.11  [1.97-4.91] | 1.24E-06 |

CHR, chromosome; SNP, rs number; BP, base position based on hg19; A1/A2, effect allele/other allele; OR, odds ratio; 95% CI, 95% confidence interval. The CIL analysis is for 225 cases and 1,654 controls (dataset 1: 155 cases and 1355 controls; dataset 2: 70 cases and 299 controls). The CIN analysis is for 43 cases and 1,654 controls (dataset 1: 18 cases and 1355 controls; dataset 2: 25 cases and 299 controls). The TRANS analysis is for the trans-ancestry GWAS meta-analysis of CIN analysis (dataset 1: 43 cases and 1,654 controls) and CIAC (dataset 2: 161 cases and 1,196 controls). rs11753309 was close to genome-wide significance.

**Supplementary Table 3:** Annotation for the lead genome-wide significant variants and their proxies

| **Lead SNP** | **Variation** | **Chr** | **BP** | **Allele** | **Name** | **Gene** | **Consequence** | **cDNA position** | **CDS position** | **Protein position** | **Amino acids** | **Codons** |
| --- | --- | --- | --- | --- | --- | --- | --- | --- | --- | --- | --- | --- |
| rs4773794 | rs11551042 | 13 | 95089735 | T | *DCT* | ENSG00000080166 | 3 prime UTR variant | 4391 | - | - | - | - |
| rs4773794 | rs16949823 | 13 | 95091202 | A | *DCT* | ENSG00000080166 | 3 prime UTR variant | 2924 | - | - | - | - |
| rs4773794 | rs1028805 | 13 | 95119269 | G | *DCT* | ENSG00000080166 | non coding transcript exon variant | 2740 | - | - | - | - |
| rs4773794 | rs11618471 | 13 | 95120324 | A | *DCT* | ENSG00000080166 | non coding transcript exon variant | 1685 | - | - | - | - |
| rs377360 | rs3701 | 14 | 23020921 | C | *TRAC* | ENSG00000229164 | 3 prime UTR variant | 798 | - | - | - | - |
| rs8024434 | rs8024434 | 15 | 73547727 | A | *AC068397.1* | ENSG00000259528 | non coding transcript exon variant | 479 | - | - | - | - |
| rs8024434 | rs12909882 | 15 | 73621946 | G | *HCN4* | ENSG00000138622 | synonymous variant | 2552 | 1558 | 520 | L | Ttg/Ctg |
| rs8024434 | rs117740286 | 15 | 73852290 | A | *REC114* | ENSG00000183324 | 3 prime UTR variant | 772 | - | - | - | - |
| rs8024434 | rs117740286 | 15 | 73852290 | A | *REC114* | ENSG00000183324 | 3 prime UTR variant | 862 | - | - | - | - |
| rs7501702 | rs1467028 | 17 | 19174874 | C | *EPN2* | ENSG00000072134 | non coding transcript exon variant | 51 | - | - | - | - |
| rs7501702 | rs1043809 | 17 | 19239432 | C | *EPN2* | ENSG00000072134 | 3 prime UTR variant | 2267 | - | - | - | - |
| rs7501702 | rs1043809 | 17 | 19239432 | C | *EPN2* | ENSG00000072134 | 3 prime UTR variant | 4068 | - | - | - | - |
| rs7501702 | rs1043809 | 17 | 19239432 | C | *EPN2* | ENSG00000072134 | 3 prime UTR variant | 4275 | - | - | - | - |
| rs7501702 | rs1043809 | 17 | 19239432 | C | *AC124066.1* | ENSG00000265263 | non coding transcript exon variant | 9 | - | - | - | - |
| rs7501702 | rs4924987 | 17 | 19247075 | G | *B9D1* | ENSG00000108641 | missense variant | 761 | 427 | 143 | Y/H | Tat/Cat |

Chr, chromosome; BP, base position based on hg19.

**Supplementary Table 4:** eQTL analyses

| **Analysis** | **SNP** | **Chr** | **BP** | **A1** | **A2** | **Probe** | **Probe_bp** | **Gene** | **p** |
| --- | --- | --- | --- | --- | --- | --- | --- | --- | --- |
| eqtl_gtex_v7/Cells_EBV-transformed_lymphocytes | rs1263663 | 14 | 23,021,770 | T | C | ENSG00000100439.6 | 23075146 | *ABHD4* | 7.49E-03 |
| eqtl_gtex_v7/Whole_Blood | rs1263663 | 14 | 23,021,770 | T | C | ENSG00000100439.6 | 23075146 | *ABHD4* | 1.88E-02 |
| eqtl_gtex_v7/Cells_EBV-transformed_lymphocytes | rs367442 | 14 | 23,021,852 | G | A | ENSG00000100439.6 | 23075146 | *ABHD4* | 3.50E-02 |
| eqtl_jenger/B_cells | rs1263663 | 14 | 23,021,770 | T | C | ENSG00000129474.11 | 23446117 | *AJUBA* | 8.58E-03 |
| eqtl_gtex_v7/Cells_EBV-transformed_lymphocytes | rs1263663 | 14 | 23,021,770 | T | C | ENSG00000129474.11 | 23446117 | *AJUBA* | 4.81E-02 |
| eqtl_jenger/B_cells | rs377360 | 14 | 23,022,276 | T | A | ENSG00000129474.11 | 23446117 | *AJUBA* | 1.36E-02 |
| eqtl_jenger/B_cells | rs393302 | 14 | 23,022,443 | C | T | ENSG00000129474.11 | 23446117 | *AJUBA* | 1.38E-02 |
| eqtl_jenger/CD8+T_cells | rs3701 | 14 | 23,020,921 | C | G | ENSG00000258643.1 | 23785311 | *BCL2L2-PABPN1* | 2.40E-02 |
| eqtl_jenger/NK_cells | rs1263663 | 14 | 23,021,770 | T | C | ENSG00000258643.1 | 23785311 | *BCL2L2-PABPN1* | 1.54E-02 |
| eqtl_jenger/Monocytes | rs1263663 | 14 | 23,021,770 | T | C | ENSG00000258643.1 | 23785311 | *BCL2L2-PABPN1* | 4.36E-02 |
| eqtl_jenger/NK_cells | rs377360 | 14 | 23,022,276 | T | A | ENSG00000258643.1 | 23785311 | *BCL2L2-PABPN1* | 5.65E-03 |
| eqtl_jenger/Monocytes | rs377360 | 14 | 23,022,276 | T | A | ENSG00000258643.1 | 23785311 | *BCL2L2-PABPN1* | 3.83E-02 |
| eqtl_jenger/NK_cells | rs393302 | 14 | 23,022,443 | C | T | ENSG00000258643.1 | 23785311 | *BCL2L2-PABPN1* | 5.63E-03 |
| eqtl_jenger/Monocytes | rs393302 | 14 | 23,022,443 | C | T | ENSG00000258643.1 | 23785311 | *BCL2L2-PABPN1* | 3.85E-02 |
| eqtl_gtex_v7/Cells_EBV-transformed_lymphocytes | rs367442 | 14 | 23,021,852 | G | A | ENSG00000100802.10 | 23467742 | *C14orf93* | 1.12E-02 |
| eqtl_gtex_v7/Liver | rs367442 | 14 | 23,021,852 | G | A | ENSG00000100802.10 | 23467742 | *C14orf93* | 3.28E-02 |
| eqtl_gtex_v7/Liver | rs424582 | 14 | 23,021,855 | A | G | ENSG00000100802.10 | 23467742 | *C14orf93* | 2.39E-02 |
| eqtl_gtex_v7/Liver | rs377360 | 14 | 23,022,276 | T | A | ENSG00000100802.10 | 23467742 | *C14orf93* | 1.29E-02 |
| eqtl_gtex_v7/Liver | rs393302 | 14 | 23,022,443 | C | T | ENSG00000100802.10 | 23467742 | *C14orf93* | 1.29E-02 |
| eqtl_muther/lcl | rs393302 | 14 | 22,092,283 | T | C | ILMN_1779095 | 22657334 | *CEBPE* | 4.44E-02 |
| eqtl_muther/lcl | rs1263663 | 14 | 22,091,610 | T | C | ILMN_1731619 | 22115814 | *DAD1* | 1.26E-02 |
| eqtl_jenger/Monocytes | rs3701 | 14 | 23,020,921 | C | G | ENSG00000129562.6 | 23045990 | *DAD1* | 1.31E-03 |
| eqtl_gtex_v7/Whole_Blood | rs3701 | 14 | 23,020,921 | C | G | ENSG00000129562.6 | 23045990 | *DAD1* | 7.78E-03 |
| eqtl_jenger/B_cells | rs3701 | 14 | 23,020,921 | C | G | ENSG00000129562.6 | 23045990 | *DAD1* | 1.94E-02 |
| eqtl_gtex_v7/Whole_Blood | rs1263663 | 14 | 23,021,770 | T | C | ENSG00000129562.6 | 23045990 | *DAD1* | 6.22E-06 |
| eqtl_jenger/Monocytes | rs1263663 | 14 | 23,021,770 | T | C | ENSG00000129562.6 | 23045990 | *DAD1* | 8.56E-03 |
| eqtl_jenger/B_cells | rs1263663 | 14 | 23,021,770 | T | C | ENSG00000129562.6 | 23045990 | *DAD1* | 1.61E-02 |
| eqtl_gtex_v7/Whole_Blood | rs367442 | 14 | 23,021,852 | G | A | ENSG00000129562.6 | 23045990 | *DAD1* | 1.05E-03 |
| eqtl_gtex_v7/Whole_Blood | rs424582 | 14 | 23,021,855 | A | G | ENSG00000129562.6 | 23045990 | *DAD1* | 1.07E-02 |
| eqtl_gtex_v7/Whole_Blood | rs377360 | 14 | 23,022,276 | T | A | ENSG00000129562.6 | 23045990 | *DAD1* | 6.73E-03 |
| eqtl_jenger/Monocytes | rs377360 | 14 | 23,022,276 | T | A | ENSG00000129562.6 | 23045990 | *DAD1* | 4.04E-02 |
| eqtl_gtex_v7/Whole_Blood | rs393302 | 14 | 23,022,443 | C | T | ENSG00000129562.6 | 23045990 | *DAD1* | 6.73E-03 |
| eqtl_jenger/Monocytes | rs393302 | 14 | 23,022,443 | C | T | ENSG00000129562.6 | 23045990 | *DAD1* | 4.08E-02 |
| eqtl_gtex_v7/Whole_Blood | rs3701 | 14 | 23,020,921 | C | G | ENSG00000100842.8 | 23830286 | *EFS* | 6.04E-03 |
| eqtl_muther/lcl | rs393302 | 14 | 22,092,283 | T | C | ILMN_1771003 | 22490734 | *HAUS4* | 8.40E-03 |
| eqtl_gtex_v7/Whole_Blood | rs3701 | 14 | 23,020,921 | C | G | ENSG00000092036.12 | 23420903 | *HAUS4* | 3.75E-02 |
| eqtl_gtex_v7/Whole_Blood | rs3701 | 14 | 23,020,921 | C | G | ENSG00000197324.4 | 23345805 | *LRP10* | 6.51E-03 |
| eqtl_jenger/NK_cells | rs3701 | 14 | 23,020,921 | C | G | ENSG00000157227.8 | 23312001 | *MMP14* | 4.90E-02 |
| eqtl_jenger/NK_cells | rs1263663 | 14 | 23,021,770 | T | C | ENSG00000157227.8 | 23312001 | *MMP14* | 4.71E-02 |
| eqtl_jenger/CD8+T_cells | rs1263663 | 14 | 23,021,770 | T | C | ENSG00000172590.14 | 23301667 | *MRPL52* | 3.42E-02 |
| eqtl_muther/lcl | rs393302 | 14 | 22,092,283 | T | C | ILMN_1784515 | 22934182 | *MYH6* | 4.52E-02 |
| eqtl_muther/lcl | rs393302 | 14 | 22,092,283 | T | C | ILMN_2324998 | 23012989 | *NGDN* | 1.61E-02 |
| eqtl_jenger/B_cells | rs1263663 | 14 | 23,021,770 | T | C | ENSG00000129460.11 | 23958984 | *NGDN* | 4.37E-02 |
| eqtl_jenger/Peripheral_blood | rs377360 | 14 | 23,022,276 | T | A | ENSG00000129460.11 | 23958984 | *NGDN* | 2.10E-02 |
| eqtl_jenger/B_cells | rs377360 | 14 | 23,022,276 | T | A | ENSG00000129460.11 | 23958984 | *NGDN* | 3.48E-02 |
| eqtl_gtex_v7/Whole_Blood | rs377360 | 14 | 23,022,276 | T | A | ENSG00000129460.11 | 23958984 | *NGDN* | 3.73E-02 |
| eqtl_jenger/Peripheral_blood | rs393302 | 14 | 23,022,443 | C | T | ENSG00000129460.11 | 23958984 | *NGDN* | 2.05E-02 |
| eqtl_jenger/B_cells | rs393302 | 14 | 23,022,443 | C | T | ENSG00000129460.11 | 23958984 | *NGDN* | 3.50E-02 |
| eqtl_gtex_v7/Whole_Blood | rs393302 | 14 | 23,022,443 | C | T | ENSG00000129460.11 | 23958984 | *NGDN* | 3.73E-02 |
| eqtl_gtex_v7/Liver | rs1263663 | 14 | 23,021,770 | T | C | ENSG00000100836.6 | 23792946 | *PABPN1* | 3.12E-02 |
| eqtl_muther/lcl | rs1263663 | 14 | 22,091,610 | T | C | ILMN_1811955 | 22464036 | *PRMT5* | 2.48E-02 |
| eqtl_jenger/CD8+T_cells | rs3701 | 14 | 23,020,921 | C | G | ENSG00000100462.11 | 23394257 | *PRMT5* | 1.94E-02 |
| eqtl_jenger/CD8+T_cells | rs1263663 | 14 | 23,021,770 | T | C | ENSG00000100462.11 | 23394257 | *PRMT5* | 1.84E-02 |
| eqtl_jenger/Monocytes | rs1263663 | 14 | 23,021,770 | T | C | ENSG00000100462.11 | 23394257 | *PRMT5* | 4.65E-02 |
| eqtl_jenger/Monocytes | rs377360 | 14 | 23,022,276 | T | A | ENSG00000100462.11 | 23394257 | *PRMT5* | 2.06E-02 |
| eqtl_jenger/CD8+T_cells | rs377360 | 14 | 23,022,276 | T | A | ENSG00000100462.11 | 23394257 | *PRMT5* | 2.82E-02 |
| eqtl_jenger/Monocytes | rs393302 | 14 | 23,022,443 | C | T | ENSG00000100462.11 | 23394257 | *PRMT5* | 2.05E-02 |
| eqtl_jenger/CD8+T_cells | rs393302 | 14 | 23,022,443 | C | T | ENSG00000100462.11 | 23394257 | *PRMT5* | 2.82E-02 |
| eqtl_gtex_v7/Whole_Blood | rs3701 | 14 | 23,020,921 | C | G | ENSG00000237054.5 | 23392385 | *PRMT5-AS1* | 3.95E-03 |
| eqtl_gtex_v7/Cells_EBV-transformed_lymphocytes | rs367442 | 14 | 23,021,852 | G | A | ENSG00000237054.5 | 23392385 | *PRMT5-AS1* | 7.71E-03 |
| eqtl_gtex_v7/Cells_EBV-transformed_lymphocytes | rs424582 | 14 | 23,021,855 | A | G | ENSG00000237054.5 | 23392385 | *PRMT5-AS1* | 7.20E-03 |
| eqtl_muther/lcl | rs393302 | 14 | 22,092,283 | T | C | ILMN_1744649 | 22569547 | *PSMB5* | 1.54E-02 |
| eqtl_jenger/CD4+T_cells | rs3701 | 14 | 23,020,921 | C | G | ENSG00000100804.14 | 23495095 | *PSMB5* | 3.52E-02 |
| eqtl_jenger/CD4+T_cells | rs1263663 | 14 | 23,021,770 | T | C | ENSG00000100804.14 | 23495095 | *PSMB5* | 1.56E-02 |
| eqtl_jenger/B_cells | rs1263663 | 14 | 23,021,770 | T | C | ENSG00000139890.5 | 23354634 | *REM2* | 4.04E-02 |
| eqtl_jenger/B_cells | rs377360 | 14 | 23,022,276 | T | A | ENSG00000139890.5 | 23354634 | *REM2* | 4.43E-02 |
| eqtl_jenger/B_cells | rs393302 | 14 | 23,022,443 | C | T | ENSG00000139890.5 | 23354634 | *REM2* | 4.42E-02 |
| eqtl_muther/lcl | rs393302 | 14 | 22,092,283 | T | C | ILMN_1653200 | 22888649 | *SLC22A17* | 3.59E-02 |
| eqtl_gtex_v7/Whole_Blood | rs3701 | 14 | 23,020,921 | C | G | ENSG00000092096.10 | 23818818 | *SLC22A17* | 2.25E-02 |
| eqtl_jenger/Peripheral_blood | rs3701 | 14 | 23,020,921 | C | G | ENSG00000092096.10 | 23818818 | *SLC22A17* | 4.43E-02 |
| eqtl_jenger/Peripheral_blood | rs1263663 | 14 | 23,021,770 | T | C | ENSG00000092096.10 | 23818818 | *SLC22A17* | 3.50E-02 |
| eqtl_jenger/Peripheral_blood | rs377360 | 14 | 23,022,276 | T | A | ENSG00000092096.10 | 23818818 | *SLC22A17* | 4.02E-02 |
| eqtl_jenger/Peripheral_blood | rs393302 | 14 | 23,022,443 | C | T | ENSG00000092096.10 | 23818818 | *SLC22A17* | 4.06E-02 |
| eqtl_jenger/NK_cells | rs3701 | 14 | 23,020,921 | C | G | ENSG00000155465.14 | 23270730 | *SLC7A7* | 1.44E-03 |
| eqtl_jenger/NK_cells | rs1263663 | 14 | 23,021,770 | T | C | ENSG00000155465.14 | 23270730 | *SLC7A7* | 2.98E-03 |
| eqtl_gtex_v7/Whole_Blood | rs424582 | 14 | 23,021,855 | A | G | ENSG00000155465.14 | 23270730 | *SLC7A7* | 3.88E-02 |
| eqtl_jenger/NK_cells | rs377360 | 14 | 23,022,276 | T | A | ENSG00000155465.14 | 23270730 | *SLC7A7* | 1.06E-02 |
| eqtl_gtex_v7/Whole_Blood | rs377360 | 14 | 23,022,276 | T | A | ENSG00000155465.14 | 23270730 | *SLC7A7* | 4.35E-02 |
| eqtl_jenger/NK_cells | rs393302 | 14 | 23,022,443 | C | T | ENSG00000155465.14 | 23270730 | *SLC7A7* | 1.06E-02 |
| eqtl_gtex_v7/Whole_Blood | rs393302 | 14 | 23,022,443 | C | T | ENSG00000155465.14 | 23270730 | *SLC7A7* | 4.35E-02 |
| eqtl_gtex_v7/Whole_Blood | rs1263663 | 14 | 23,021,770 | T | C | ENSG00000092068.14 | 23623693 | *SLC7A8* | 3.33E-02 |
| eqtl_jenger/CD8+T_cells | rs377360 | 14 | 23,022,276 | T | A | ENSG00000229164.5 | 23018772 | *TRAC* | 4.72E-02 |
| eqtl_jenger/CD8+T_cells | rs393302 | 14 | 23,022,443 | C | T | ENSG00000229164.5 | 23018772 | *TRAC* | 4.69E-02 |
| eqtl_jenger/CD8+T_cells | rs377360 | 14 | 23,022,276 | T | A | ENSG00000211789.2 | 22356359 | *TRAV12-2* | 4.87E-02 |
| eqtl_jenger/CD8+T_cells | rs393302 | 14 | 23,022,443 | C | T | ENSG00000211789.2 | 22356359 | *TRAV12-2* | 4.88E-02 |
| eqtl_jenger/CD4+T_cells | rs377360 | 14 | 23,022,276 | T | A | ENSG00000211791.2 | 22386647 | *TRAV13-2* | 4.49E-02 |
| eqtl_jenger/CD4+T_cells | rs393302 | 14 | 23,022,443 | C | T | ENSG00000211791.2 | 22386647 | *TRAV13-2* | 4.46E-02 |
| eqtl_jenger/Peripheral_blood | rs3701 | 14 | 23,020,921 | C | G | ENSG00000211800.3 | 22509119 | *TRAV20* | 4.17E-02 |
| eqtl_jenger/Peripheral_blood | rs1263663 | 14 | 23,021,770 | T | C | ENSG00000211800.3 | 22509119 | *TRAV20* | 3.06E-02 |
| eqtl_gtex_v7/Whole_Blood | rs367442 | 14 | 23,021,852 | G | A | ENSG00000211800.3 | 22509119 | *TRAV20* | 8.48E-03 |
| eqtl_gtex_v7/Whole_Blood | rs424582 | 14 | 23,021,855 | A | G | ENSG00000211800.3 | 22509119 | *TRAV20* | 1.09E-02 |
| eqtl_gtex_v7/Whole_Blood | rs377360 | 14 | 23,022,276 | T | A | ENSG00000211800.3 | 22509119 | *TRAV20* | 1.02E-02 |
| eqtl_jenger/Peripheral_blood | rs377360 | 14 | 23,022,276 | T | A | ENSG00000211800.3 | 22509119 | *TRAV20* | 4.52E-02 |
| eqtl_gtex_v7/Whole_Blood | rs393302 | 14 | 23,022,443 | C | T | ENSG00000211800.3 | 22509119 | *TRAV20* | 1.02E-02 |
| eqtl_jenger/Peripheral_blood | rs393302 | 14 | 23,022,443 | C | T | ENSG00000211800.3 | 22509119 | *TRAV20* | 4.55E-02 |
| eqtl_gtex_v7/Whole_Blood | rs3701 | 14 | 23,020,921 | C | G | ENSG00000211805.1 | 22573871 | *TRAV24* | 1.39E-02 |
| eqtl_gtex_v7/Whole_Blood | rs1263663 | 14 | 23,021,770 | T | C | ENSG00000211805.1 | 22573871 | *TRAV24* | 3.76E-02 |
| eqtl_gtex_v7/Whole_Blood | rs367442 | 14 | 23,021,852 | G | A | ENSG00000211812.1 | 22670869 | *TRAV26-2* | 2.69E-02 |
| eqtl_gtex_v7/Whole_Blood | rs424582 | 14 | 23,021,855 | A | G | ENSG00000211812.1 | 22670869 | *TRAV26-2* | 3.54E-02 |
| eqtl_gtex_v7/Whole_Blood | rs377360 | 14 | 23,022,276 | T | A | ENSG00000211809.2 | 22616268 | *TRAV27* | 4.47E-02 |
| eqtl_gtex_v7/Whole_Blood | rs393302 | 14 | 23,022,443 | C | T | ENSG00000211809.2 | 22616268 | *TRAV27* | 4.47E-02 |
| eqtl_jenger/CD8+T_cells | rs3701 | 14 | 23,020,921 | C | G | ENSG00000259092.1 | 22636604 | *TRAV30* | 3.98E-02 |
| eqtl_jenger/CD8+T_cells | rs1263663 | 14 | 23,021,770 | T | C | ENSG00000259092.1 | 22636604 | *TRAV30* | 2.59E-02 |
| eqtl_jenger/CD8+T_cells | rs377360 | 14 | 23,022,276 | T | A | ENSG00000259092.1 | 22636604 | *TRAV30* | 2.89E-02 |
| eqtl_jenger/CD8+T_cells | rs393302 | 14 | 23,022,443 | C | T | ENSG00000259092.1 | 22636604 | *TRAV30* | 2.86E-02 |
| eqtl_jenger/NK_cells | rs1263663 | 14 | 23,021,770 | T | C | ENSG00000211787.1 | 22320959 | *TRAV8-3* | 2.76E-02 |
| eqtl_jenger/NK_cells | rs3701 | 14 | 23,020,921 | C | G | ENSG00000211829.2 | 22933351 | *TRDC* | 2.56E-03 |
| eqtl_jenger/NK_cells | rs1263663 | 14 | 23,021,770 | T | C | ENSG00000211829.2 | 22933351 | *TRDC* | 2.45E-02 |
| eqtl_jenger/NK_cells | rs377360 | 14 | 23,022,276 | T | A | ENSG00000211829.2 | 22933351 | *TRDC* | 1.21E-03 |
| eqtl_jenger/NK_cells | rs393302 | 14 | 23,022,443 | C | T | ENSG00000211829.2 | 22933351 | *TRDC* | 1.19E-03 |
| eqtl_jenger/NK_cells | rs3701 | 14 | 23,020,921 | C | G | ENSG00000256590.2 | 22938361 | *TRDV3* | 5.04E-03 |
| eqtl_jenger/NK_cells | rs1263663 | 14 | 23,021,770 | T | C | ENSG00000256590.2 | 22938361 | *TRDV3* | 4.78E-03 |
| eqtl_jenger/NK_cells | rs377360 | 14 | 23,022,276 | T | A | ENSG00000256590.2 | 22938361 | *TRDV3* | 1.29E-02 |
| eqtl_jenger/NK_cells | rs393302 | 14 | 23,022,443 | C | T | ENSG00000256590.2 | 22938361 | *TRDV3* | 1.29E-02 |
| eqtl_jenger/B_cells | rs377360 | 14 | 23,022,276 | T | A | ENSG00000136367.12 | 24007733 | *ZFHX2* | 4.82E-02 |
| eqtl_jenger/B_cells | rs393302 | 14 | 23,022,443 | C | T | ENSG00000136367.12 | 24007733 | *ZFHX2* | 4.85E-02 |
| eqtl_muther/lcl | rs7850169 | 9 | 24,142,518 | C | A | ILMN_1769369 | 23748083 | *ELAVL2* | 4.82E-02 |

eqtl_gtex_v7: the Genotype-Tissue Expression project release V7; eqtl_jenger: the Japanese Encyclopedia of Genetic Associations by Riken; eqtl_muther: the Multiple Tissue Human Expression Resource.

**Supplementary Table 5:** GWAVA scores for the lead genome-wide significant variants

| **SNP** | **Chromosome** | **Position** | **Region score** | **TSS score** | **Unmatched score** |
| --- | --- | --- | --- | --- | --- |
| rs4773794 | 13 | 95121488 | 0.41 | 0.41 | 0.59 |
| rs8024434 | 15 | 73547727 | 0.46 | 0.20 | 0.49 |
| rs7501702 | 17 | 19293727 | 0.38 | 0.28 | 0.24 |
| rs377360 | 14 | 23022276 | 0.24 | 0.26 | 0.23 |
| rs9808117 | 2 | 197110855 | 0.40 | 0.24 | 0.20 |
| rs373695 | 6 | 6184352 | 0.25 | 0.28 | 0.10 |
| rs10512698 | 3 | 127965570 | 0.35 | 0.15 | 0.09 |
| rs73482673 | 9 | 24121611 | 0.32 | 0.11 | 0.03 |
| rs116982346 | 3 | 108586107 | 0.23 | 0.14 | 0.00 |

**Supplementary Table 6:** List of oligonucleotides used for luciferase reporter vectors construction

| **SNP** | **Allele** | **Forward sequence (5'-3')** | **Reverse sequence (5'-3')** |
| --- | --- | --- | --- |
| rs9808117 | C | cGAAAGGCATAATTTGCAACACGAAGGAACATc | TCGAGATGTTCCTTCGTGTTGCAAATTATGCCTTTCGGTAC |
|  | T | cGAAAGGCATAATTTGTAACACGAAGGAACATc | TCGAGATGTTCCTTCGTGTTACAAATTATGCCTTTCGGTAC |
| rs4773794 | G | cGACTAAGCACTAGAGGGTTTCCTAGACACATc | TCGAGATGTGTCTAGGAAACCCTCTAGTGCTTAGTCGGTAC |
|  | A | cGACTAAGCACTAGAGAGTTTCCTAGACACATc | TCGAGATGTGTCTAGGAAACTCTCTAGTGCTTAGTCGGTAC |
| rs377360 | A | cAGATTTTGGAGCATTACCAATTTCAGATTTTc | TCGAGAAAATCTGAAATTGGTAATGCTCCAAAATCTGGTAC |
|  | T | cAGATTTTGGAGCATTTCCAATTTCAGATTTTc | TCGAGAAAATCTGAAATTGGAAATGCTCCAAAATCTGGTAC |
| rs8024434 | A | cGCATACCTGGTAAAAATGGATCCACATGTGTc | TCGAGACACATGTGGATCCATTTTTACCAGGTATGCGGTAC |
|  | C | cGCATACCTGGTAAAACTGGATCCACATGTGTc | TCGAGACACATGTGGATCCAGTTTTACCAGGTATGCGGTAC |
| rs7501702 | G | cAGAATCCATTCCTGGGGCGATGGTGACCCCAc | TCGAGTGGGGTCACCATCGCCCCAGGAATGGATTCTGGTAC |
|  | A | cAGAATCCATTCCTGGAGCGATGGTGACCCCAc | TCGAGTGGGGTCACCATCGCTCCAGGAATGGATTCTGGTAC |
